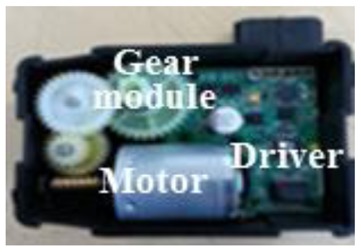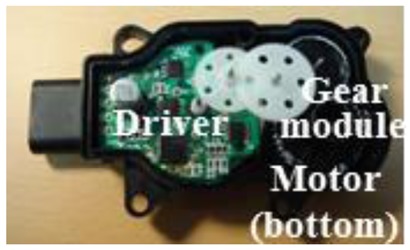# Development of a Micro-Step Voltage-Fed Actuator with a Novel Stepper Motor for Automobile AGS Systems

**DOI:** 10.3390/s140508026

**Published:** 2014-05-05

**Authors:** Se-Hyun Rhyu, Jeong-Jong Lee, Bon-Gwan Gu, Byung-Dae Choi, Jung-Hyuk Lim

**Affiliations:** 1 Korea Electronics Technology Institute, 203-101, Bucheon TP 192 Yakdae-dong, Wonmi-gu, Bucheon-si, Gyeonggi-do 420-140, Korea; E-Mail: leejj@keti.re.kr; 2 School of Energy Engineering, Kyungpook National University, Daegu 702-701, Korea; E-Mail: bggu@knu.ac.kr; 3 O&K Technology Co. Ltd., Seoul 152-769, Korea; E-Mails: bdchoi@onktech.com (B.-D.C.); jhlim@onktech.com (J.-H.L.)

**Keywords:** stepper motor, active grill shutter (AGS), micro-step, actuator, finite element analysis (FEA), cogging torque, space vector PWM

## Abstract

This paper presents an improved micro-step voltage-fed actuator for an automobile active grill shutter (AGS) system. A novel structured stepper motor, which contains both the main and auxiliary teeth in the stator, is proposed for the actuator. In a normal permanent magnet (PM) motor coils are generally wound on all the stator teeth, however, in the proposed motor, the winding is only on the main teeth. Because of the absence of coils in the auxiliary teeth, the proposed stepper motor possesses the following advantages: simple structure, lighter weight, smaller volume, and less time consumption. The unique auxiliary poles in the stepper motor supply the flux path to increase the step resolution even without any coils. The characteristics of the proposed stepper motor were investigated using finite element analysis. In particular, the effect of the magnetization distribution of the PM on the motor performance was investigated during the analysis. Cogging torque, which causes noise and vibration issues, was minimized by the tooth-shape optimization. In addition, a micro-step voltage-fed algorithm was implemented for a high-resolution position control. By employing a current close to a sine wave using space vector pulse-width modulation, a high-quality current waveform with a high resolution was obtained. Finally, the proposed prototype was fabricated, and the cogging torque, back-electromotive force, and current characteristics were measured by mounting the prototype on the AGS system. Both the analysis and experimental results validate the performance improvement from the proposed motor and its possible application for the flap control of the AGS system.

## 1. Introduction

Recently, active grill shutters (AGSs) have been put in the spotlight as actuating modules for fuel economy in automobiles. The actuating module controls the air flap of an AGS system because it can reduce air resistance and improve the cooling effect, which conserves energy during driving by allowing the automobile to run at high speed and thus longer distance. Therefore, the electrical performance of the actuator, which controls the flap angle of the AGS system, is very important. In particular, a light and slim actuator design is required to enhance the fuel economy, and a network-driver-integrated structure design is needed to communicate with the car engine control unit. A gear module with a high deceleration ratio using a slim motor is also required to provide more strength. Thus, the motor should have a very simple structure for it to be installed into the AGS system driving actuator and for proper arrangement of the other parts. Harmful factors, e.g., cogging torque, which cause vibration or decrease the angle control precision of the actuator when driven, should be reduced to the minimum.

Generally, brush-type DC motors are used in AGS systems; however, they reduce the AGS performance because of their low efficiency and weak controllability. On the other hand, the stepper motor is suitable as a control device, such as in the AGS system that requires maximum angular accuracy, because it is capable of open loop control [1]. The step-motor controller can smoothly perform positioning control. To realize smooth positioning, the micro-step mode that uses a pulse-width modulation (PWM) approach is an excellent selection [2,3]. However, designing a small-size slim stepper motor with high performance is difficult because of the complex winding structure and the difficulty of multi-polarization in small-size PMs. In the present paper, a newly structured PM-type stepper motor with auxiliary poles and partial winding is proposed for an automobile AGS system. In addition, we analyze the magnetic characteristics and compare the result with the general-type model using finite element analysis (FEA) to account for the magnetization distribution of the PM in the magnetizing yoke. The simulation and experimental results show that the proposed model can be applied in the AGS system.

## 2. AGS Actuator and Characteristic Analysis

### 2.1. Actuator with Novel Stepper Motor for AGS System

**Figure 1.**
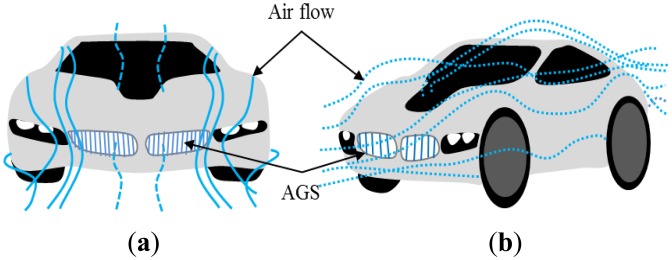
Air-flow concept in AGS. (**a**) Flap *ON*. (**b**) Flap *OFF*.

**Figure 2.**
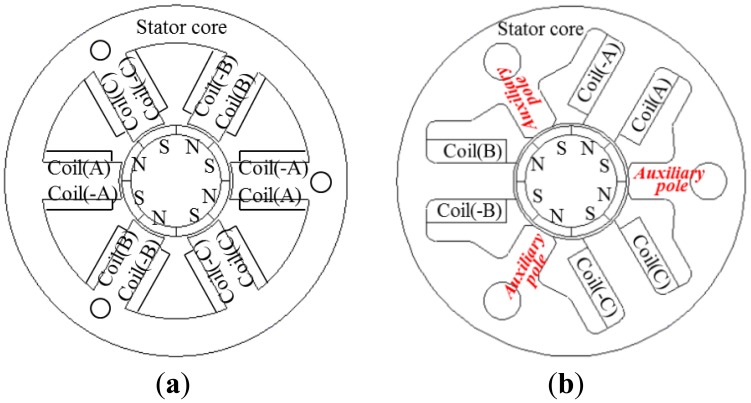
Stator-core shape of the AGS. (**a**) General motor type. (**b**) Proposed motor.

Figure 1 shows the air-flow concept according to the flap *ON* and *OFF* in an automobile AGS system. The AGS system has an air flap and an actuating module that consists of a precision motor, a driver, and a gear stack. If an automobile is driven at high speed, the flap is closed by the actuating module. Figure 2 shows the structure of the general type and the proposed three-phase PM-type stepper motor models. The stator consists of three-phase exciting coils and a core with a typical tooth shape. However, relative to the structure among the PM, coil, and tooth width, the coil turns are restricted to reduce the overall PM size following the decrease in the slot width. Furthermore, we confirm that the winding connection and the manufacturing process of the motor are complex, as shown in Figure 2a. In this paper, we propose a slim-type stepper motor with a novel structure with auxiliary poles. Although the proposed model consists of the same parts as the general-type model, it has a different structure. The proposed model is composed of three poles with coils, which can provide the same step resolution because it has three auxiliary poles that offer a magnetic coupling path, as shown in Figure 2b. In addition, the proposed model has an extremely simple winding connection and manufacturing process compared with the general-type model.

The magnetic characteristics of the proposed motor are analyzed by FEA to account for the PM magnetization. Table 1 lists the specifications of the proposed model.

### 2.2. Characteristic Calculation Using FEA

**Figure 3.**
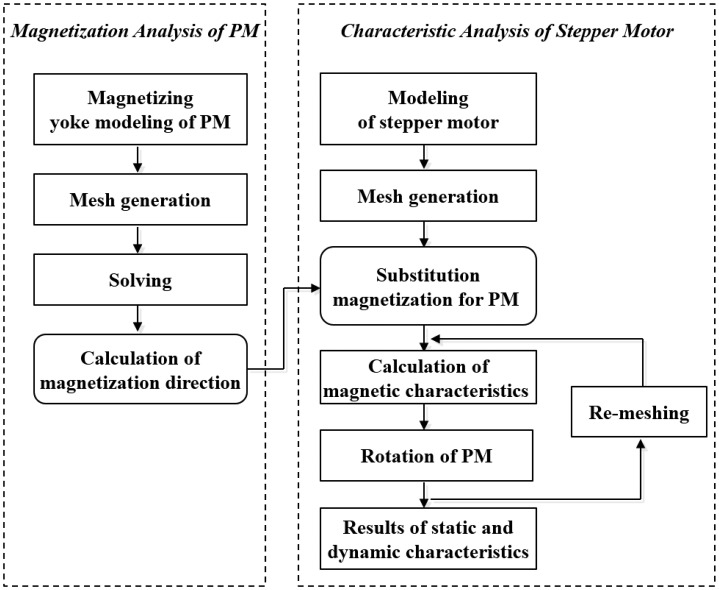
Flowchart of the magnetic characteristic calculation of the stepper motor.

Figure 3 shows the flowchart of the static and dynamic characteristic analysis using the FE method directly. First, the distribution of the PM magnetic flux density is computed by analyzing the magnetizing yoke. Using the distribution of the magnetization in the PM region, we calculate the magnetic characteristics of the stepper motor [4]. The fundamental equation for the PM magnetic field using the magnetic vector potential **A** is:

(1) $$\text{rot}(\upsilon \mathrm{rot}A)={\text{J}}_{o}+\upsilon_{o}\mathrm{rot}M$$

where *υ* is the reluctivity, *υ_o_* is the reluctivity of vacuum, **J***_o_* is the exciting current density, and **M** is magnetization of the PM.

**Figure 4.**
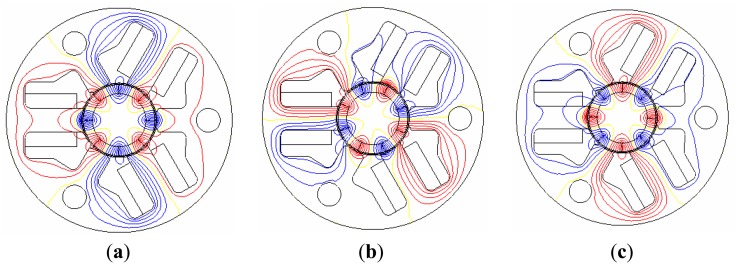
Flux line according to the rotor position: (**a**) 0°, (**b**) 22.5°, and (**c**) 45°.

**Figure 5.**
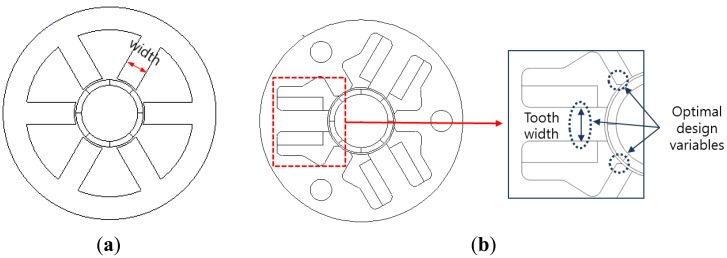
Design variables for the cogging-torque reduction: (**a**) initial model and (**b**) proposed model.

**Figure 6.**
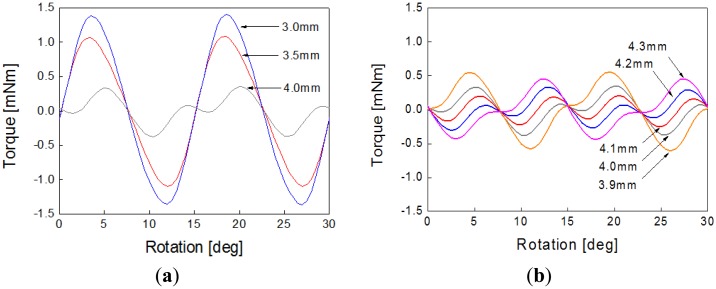
Simulation results of the cogging torque according to the tooth shape: (**a**) initial model and (**b**) proposed model.

**Figure 7.**
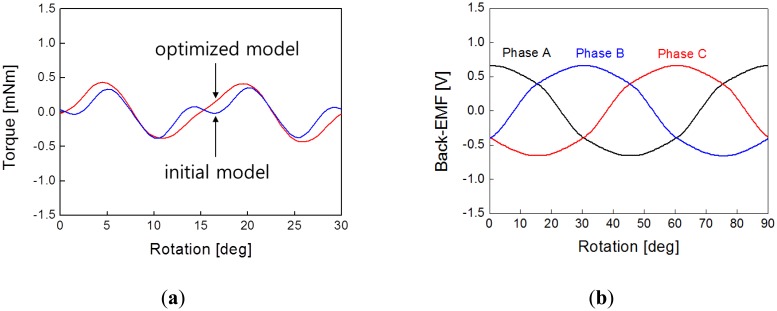
Simulation results of the optimized model: (**a**) cogging torque and (**b**) back-EMF (720 rpm).

Figure 4 shows the flux line distribution according to the rotor position, which shows that the auxiliary poles also play a key role in the entire magnetic circuit. Figure 5a shows the initial model of a PM-type stepper motor with a normal stator structure. The proposed model is developed on the basis of this model, which has a magnetic circuit in the auxiliary poles, as shown in Figure 5b. In this study, the tooth width and shape are selected as design variables to minimize the cogging torque, which is an important factor in the noise and vibration of the stepper motor. The tooth width and shape acutely affect the main flux and are major factors that determine the size of the coil winding assembly. Figure 6a shows the analysis results of the cogging torque within the range of the tooth width with a 0.5-mm interval in the initial model. Figure 6b shows the cogging torque of the proposed model with a 0.1-mm interval on the basis of the model with a 4-mm tooth width. Here, the amplitude and period of the cogging torque are varied by varying the tooth width, where a minimum value is obtained at a 4.1-mm tooth width. Thus, the model with a 4.1-mm tooth width is considered as the optimized model, which has a cogging torque of 0.45 mN·m, as shown in Figure 7a. Figure 7a shows the evaluation results of the cogging torque, which are the minimized and sinusoidal waveforms obtained from the tooth width and shape variation. For cogging torque reduction and coil inserting, we have cut the tooth shoe using trial and error.

We must note that the characteristic of the cogging torque is a very important factor for micro-step resolution. In addition, Figure 7b shows the simulation result of the back electromotive force (back-EMF) waveform at 720 rpm, which is a sinusoidal waveform. Thus, we can predict that the cogging torque, which is an important factor to determine, can be possibly reduced by further experiments by considering the exact magnetization distribution of the PM. If the magnetization distribution of the PM is not accurately considered, then many errors would appear on the static and dynamic simulation results. The cogging-torque characteristic factor determines whether the cogging torque can have a minimized amplitude and an optimized sinusoidal waveform by tooth-width and shape optimization. Figure 7b shows that the simulation result waveform of the back-EMF is almost sinusoidal at 720 rpm. Thus, the cogging torque can be possibly reduced. The cogging torque is an important parameter in determining whether the PM is accurately manufactured and its magnetization accurately distributed. Inaccurate PM magnetization distribution would lead to many errors in the static and dynamic simulation results.

## 3. Experimentation

**Figure 8.**
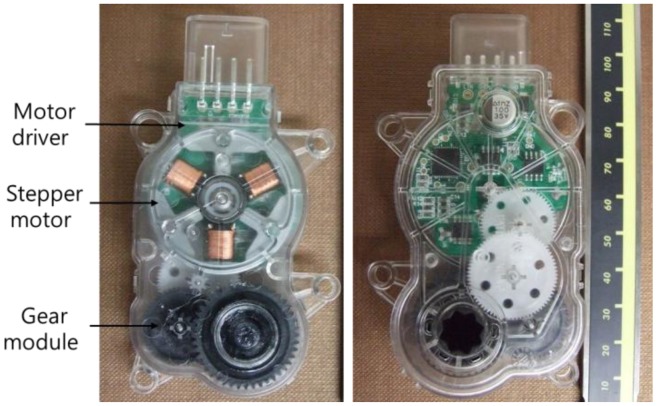
Actuator prototypes of the AGS system: (**a**) upper side and (**b**) lower side.

Figure 8 shows the prototypes of the actuating module composed of a stepper motor, a motor driver, and a gear module. The figure shows that the stepper motor, a key driver of the actuator, simply consists of the stator, which consists of the coils, and the rotor, which is a permanent magnet. In particular, while the stator core has six teeth, it has a three-phase winding structure with only three coil groups. The coil of each phase has a structure assembly that allows it to be conveniently plugged at the inner side of the stator. As a result, the torque, speed, and rotational position characteristics of the rotor are output through the gear module to the final shaft of the actuator.

**Figure 9.**
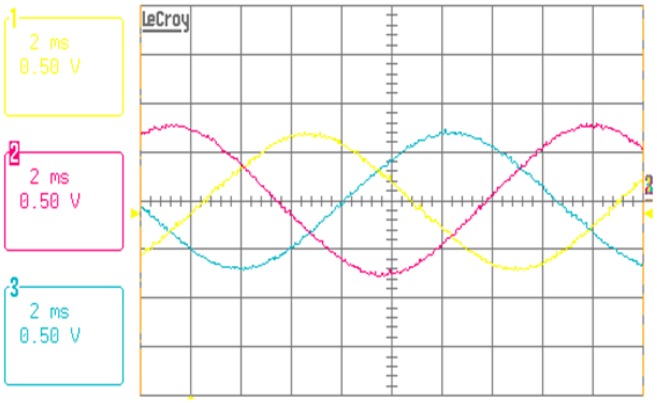
Experimental results of the back-EMF measurement (720 rpm).

The experimental result of the back-EMF is shown in Figure 9. The amplitude of the back-EMF is approximately 0.69 V. The figure shows that the simulation and experimental results have very similar values. From these results, we can confirm that the PM magnetization is accurately considered in the FEA.

**Figure 10.**
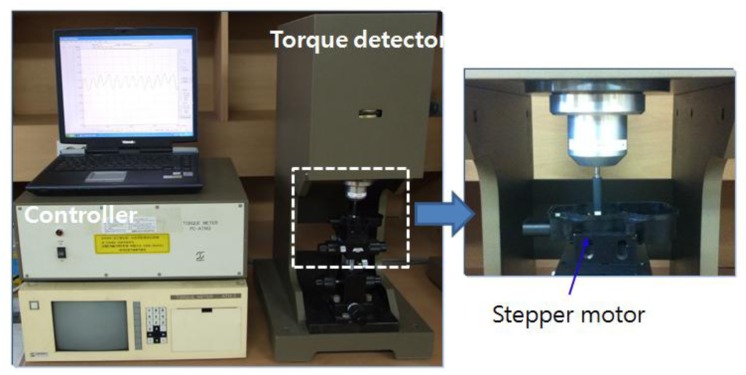
Experimental setup for the cogging-torque measurement.

**Figure 11.**
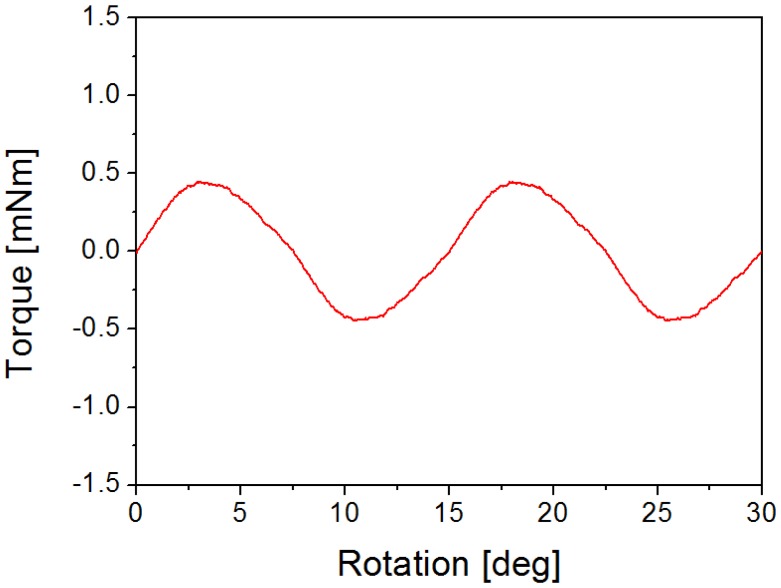
Experimental results of the cogging-torque measurement.

Figure 10 shows the experimental setup to measure the characteristics of the cogging torque of the stepper motor using a torque meter (ATM-5MN, SUGAWARA, Tokyo, Japan). After the stepping motor is connected to the torque meter, the torque is measured according to the rotor angle. The measurement of the cogging-torque waveform is shown in Figure 11. Compared with the calculated value of the holding torque shown in Figure 7a, the amplitude of the measured torque is 0.44 mN·m. Both results are very similar and have only a slight error. Also, Table 2 shows the characteristic comparison between developed prototypes and traditional actuator. We can see that the developed actuator has a small size and very light weight compare with commercial model.

The PWM method, which controls the magnitude of the voltage, is generally used to control the stepper motor. On the other hand, sinusoidal pulse width modulation (SPWM) is widely used, which simply compares the output voltage and the sine table. The present study used the space vector PWM, which has the advantage of higher differential output voltage, and not the SPWM method to control the voltage. The space vector PWM has an approximately 15% higher voltage magnitude than the SPWM. It can make the voltage differences among the motor lines appear as a sine wave only and has the advantage of being able to reduce the switching loss because it always prevents the occurrence of one-phase switch change [5].

**Figure 12.**
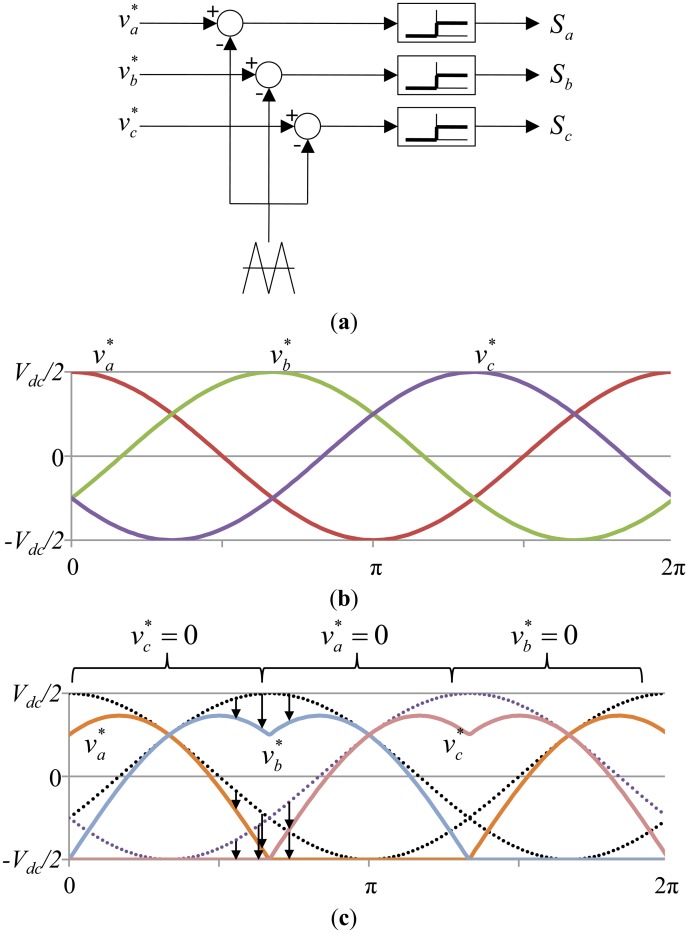
Comparison of the input voltage control methods for the stepper motor. (**a**) Signal block diagram of the PWM. (**b**) Modulation waveforms of SPWM. (**c**) Modulation waveforms of Space vector PWM.

Figure 12 shows the comparison of the PWM duty generation methods. $\nu_{a}^{*}$, $\nu_{b}^{*}$ and $\nu_{c}^{*}$ are the output voltage commands, *S_a_*, *S_b_* and *S_c_* are switch *ON* and *OFF* signal, respectively. *V_dc_* denotes the controller input DC voltage. Figure 12a shows the signal block diagram of the general PWM utilizing the triangle intersection technique. Figure 12b shows the general SPWM method based voltage commands, which generates a PWM signal by simply comparing the output voltage from the sine table with the triangular wave, and the three-phase switch continuously alternates between *ON* and *OFF*. In contrast, the differences among the voltages are maintained, and the phase with the lowest output voltage is reduced to zero, which stops the one-phase switching; thus, the margin of the output voltages is obtained by the space vector PWM, as shown in Figure 12c.

**Figure 13.**
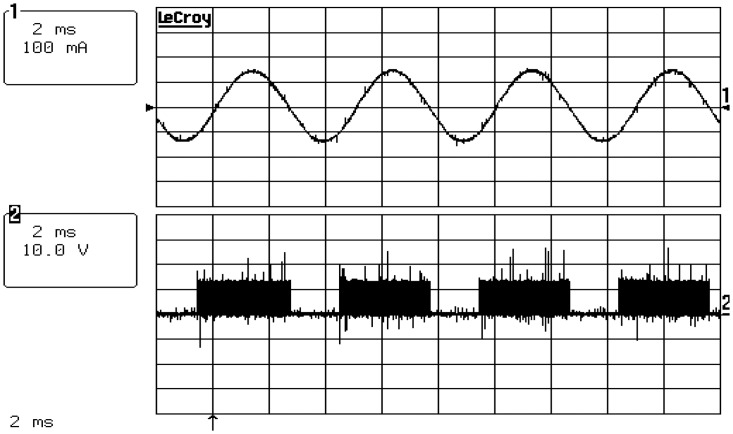
Experimental results of the current and voltage waveforms (3000 rpm, 1.3 mNm).

Figure 13 shows the measured voltage and current waveforms of the fabricated actuator prototypes with a micro-step voltage fed control at 3000 rpm and a 1.3-mN·m load. Figure 13 shows that a sinusoidal current waveform with very little harmonic components is generated. In other words, we can conclude that it is very advantageous in terms of vibration or noise when the motor is operated. One cycle of the current waveform has a 180-step resolution, and we confirm the possibility for precision position control owing to the high resolution.

We determine that this favorable result is due to the cogging-torque-reduction design and the sinusoidal back-EMF waveforms of the proposed stepper motor. The precision position control of the flap in the AGS system is very important to control the optimum amount of air flow into the car, and we confirm that the stepper motor and micro-step voltage-fed control method proposed in this paper can be very useful in the flap control of the AGS system.

## 4. Conclusions

In this paper, a slim-type stepper motor with a novel structure has been proposed for the automobile AGS system. The shape design of the initial model was changed to a common structure that considers the ease and convenience of production. In particular, a structure that can reduce the number of coils using auxiliary poles has been proposed. The validity of the structural design has been confirmed by the adequacy of the back-EMF waveform and the path of the flux line at each rotor position. Here, we confirmed that the auxiliary pole plays an adequate role as a common pole in the construction of a magnetic circuit. The cogging torque was also minimized by the tooth-shape optimization design, which improved the position-control characteristic. The FEA result that considers the magnetization distribution of the PM was used for the characteristic analysis method. We confirmed through experiments that the cogging torque and the back-EMF characteristics agree well with the analyzed values, and the actuator fabricated with the proposed motor exhibits a high-resolution behavior by measuring the voltage and current waveforms under the micro-step control condition using the space vector PWM.

On the basis of the smaller actuators and high-resolution position, we have developed a novel actuator structure that is easy to manufacture. Thus, we confirmed that our proposed novel stepper motor with partial winding is very useful for actuators used to drive a car AGS system. However, further study related to the position control responsiveness according to the load variation is necessary.

## Figures and Tables

**Table 1.** Specifications of the proposed stepper motor.

**Item**	**Value**	**Unit**
Number of phase	3	phases
Outer diameter of stator	38.0	mm
Length of stator	4.0	mm
PM type	Bonded NdFeB	-
Number of poles	8	poles

**Table 2.** Comparison of actuator (commercial model *vs.* developed model).

**Item**	**Commercial Product**	**Developed Prototype**
Type of motor	Brush type DC	Stepper
Size of motor	(*Diameter*) 27.7 mm × (*Length*) 58 mm	(*Diameter*) 38.0 mm × (*Height*) 4.0 mm
Size of actuator	(*Length*) 94 mm × (*Width*) 77 mm × (*Height*) 31 mm	(*Length*) 99.3 mm × (*Width*) 56.2 mm × (*Height*) 23.7 mm
Control method	Open and Shot ( *ON* or *OFF* )	Position control
Weight of actuator	285 g	75 g
Photo